# Beyond cytotoxic T cells: reprogrammed regulatory T cells help facilitate response to dual checkpoint blockade

**DOI:** 10.1002/1878-0261.70076

**Published:** 2025-06-15

**Authors:** Tullia C. Bruno, Anthony R. Cillo

**Affiliations:** ^1^ UPMC Hillman Cancer Center University of Pittsburgh PA USA; ^2^ Cancer Immunology and Immunotherapy Program University of Pittsburgh PA USA; ^3^ Department of Immunology University of Pittsburgh PA USA; ^4^ Center for Systems Immunology University of Pittsburgh PA USA

**Keywords:** cancer immunology, immuno‐oncology, immunotherapy, regulatory T cells, Treg fragility, Treg stability

## Abstract

Combination immunotherapies have entered the treatment armamentarium of oncology, but important knowledge gaps remain in our understanding of how these therapeutics work. A recent study by Rolig, Peng, and colleagues has shed new light on how dual blockade of PD1 and LAG3 enhances antitumor immunity. The authors first interrogated LAG3 expression on T cells across murine tumor models, classifying the models as LAG3^hi^ or LAG3^lo^. Next, they found that LAG3^hi^ models were unresponsive to anti‐PD1 alone but responsive to combination therapy with anti‐PD1 + anti‐LAG3. Surprisingly, the response to anti‐PD1 + anti‐LAG3 in LAG3^hi^ models was associated with reprogramming of CD4^+^ regulatory T cells (Treg) from the canonically immunosuppressive state to an inflammatory state characterized by loss of expression of the transcription factor Foxp3 and upregulation of transcription factor Tbet. Importantly, an analogous reprogrammed Treg state was associated with response to anti‐PD1 + anti‐LAG3 and longer overall survival in patients with metastatic melanoma. This work highlights the importance of cells beyond cytotoxic CD8^+^ T cells as drivers of response to immunotherapy and sets the stage for subsequent mechanistic and translational studies.

## Graphical abstract

Rolig, Peng, and colleagues have shed new light on how dual blockade of PD1 and LAG3 enhances antitumor immunity. They found that response to immunotherapy with anti‐PD1 + anti‐LAG3 was associated with reprogramming of canonically suppressive CD4^+^ regulatory T cells to an inflammatory state. These findings highlight the importance of cell populations beyond cytotoxic CD8^+^ T cells as contributors to efficacious immunotherapy.

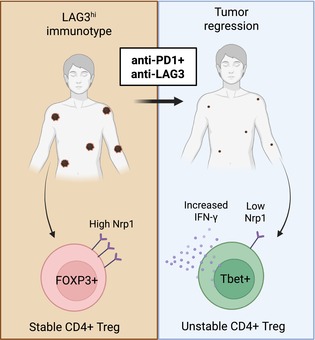

**Abbreviations**
IO: immunotherapy
Treg: CD4^+^ regulatory T cells
PBMC: peripheral blood mononuclear cells

Immunotherapy (IO) is now routinely used for the treatment of many solid tumors, but not all patients garner a long‐term therapeutic benefit. Thus, combinatorial therapeutics are now being targeted to bolster response rates [1, 2]. Two Food and Drug Administration‐approved regimens that target inhibitory pathways are anti‐PD1/anti‐CTLA4 or anti‐PD1/anti‐LAG3; other regimens and alternative dosing strategies are being actively pursued in various stages of clinical trials. However, despite the strong interest in and clinical efficacy of combination immunotherapies, much remains to be elucidated regarding the biological mechanisms that are associated with clinical benefit in patients. Understanding the mechanisms involved in response and resistance would provide two benefits: (a) insight into the fundamental biology of inhibitory receptor‐mediated suppression of immune function and (b) rational design of optimal combinatorial therapeutic strategies. Ultimately, a better understanding of the mechanisms governing IO response would allow for the development of more efficacious immunotherapeutic strategies.

Rolig, Peng, and colleagues recently added to the growing body of literature on the mechanisms associated with IO response following combination therapy with anti‐PD1 and anti‐LAG3 [3]. This work builds on their previous observation that response to anti‐PD1 monotherapy could be delineated by two peripheral blood immunotypes in patients with urothelial cancer or metastatic melanoma: LAG3^hi^ and LAG3^lo^, with the latter being responsive to anti‐PD1 monotherapy and the former being resistant [4]. To elucidate the mechanisms associated with these immunotypes, the authors evaluated 10 tumor models across two different strains of mice to recapitulate the previously observed human immunotypes. From these studies, mice bearing CT26 and B16F10 tumors resembled the LAG3^hi^ immunotype and those bearing MCA‐205 resembled the LAG3^lo^ immunotype. A key observation surfaced with depletion of either CD8^+^ or CD4^+^ T cells in conjunction with anti‐PD1 + anti‐LAG3 therapy; as expected, depletion of CD8^+^ T cells abrogated the effect of IO, but surprisingly, depletion of CD4^+^ T cells improved survival in the murine CT26 model. This observation suggested that CD4^+^ T cells play a deleterious role in the context of the LAG3^hi^ immunotype and warranted further dissection.

Determining how CD4^+^ T cells contribute to IO resistance was subsequently dissected via interrogation of murine models and patient specimens. Through single‐cell RNAseq experiments and high‐dimensional flow cytometry, responders to combination therapy contained a population of CD4^+^ regulatory T cells (Treg) that upregulated the effector cytokine IFN‐y and the type 1 helper T‐cell‐related transcription factor Tbet with downregulation of Nrp1 (a key driver of Treg stability [5, 6]; Fig. 1). From these data, the authors hypothesized that Treg may be undergoing conversion from stable and suppressive Treg to fragile or ex‐Treg. These Treg states have been described across multiple systems, with stable Treg being the canonical immunosuppressive population, fragile Treg retaining expression of Foxp3 but gaining secretion of IFN‐γ [7], and unstable or ex‐Treg having lost Foxp3 expression entirely and adopted a state similar to conventional helper T cells [8]. Additional experiments were required to identify the Treg state in the responders to combination IO.

**Fig. 1:**
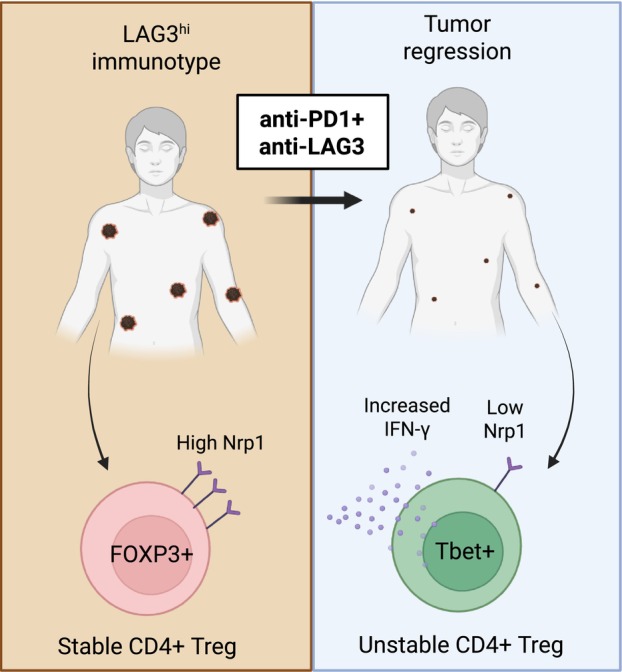
Anti‐PD1 + anti‐LAG3 therapy converts stable CD4^+^ T regulatory cells (Treg) to unstable Treg in murine models and patients with a LAG3^hi^ immunotype. Some murine tumor modules and a subset of patients can be classified as having a LAG3^hi^ immunotype. Treg are canonically an immunosuppressive population that express high levels of the transcription factor FOXP3 and high levels of Nrp1 on the cell surface. However, following combination immunotherapy with anti‐PD1 + anti‐LAG3 in both murine models and patients with metastatic melanoma, Treg are reprogrammed to an inflammatory state characterized by low expression of FOXP3 and Nrp1 and high expression of the transcription factor Tbet, termed ‘unstable’ Treg. These unstable Treg are associated with enhanced antitumor immunity and improved overall survival in melanoma patients.

To address this question, the authors employed a genetic lineage tracing experiment, where Foxp3 expression was reported by GFP and simultaneously induced permanent expression of YFP. This experiment revealed that a subpopulation of Treg loses Foxp3 expression (that is, are YFP+ GFP–) in mice that respond to combination IO. Previous work has identified a population of Foxp3‐expressing fragile Treg responsible for IO response [7]; this work suggests that simultaneous blockade of LAG3 and PD1 destabilizes Foxp3 expression and leads to ex‐Treg. Finally, the authors performed high‐dimensional flow cytometry in peripheral blood mononuclear cells (PBMC) from a large cohort of metastatic melanoma patients that received anti‐PD1 + anti‐LAG3. Like the mouse model, patients that responded to combination therapy had a subpopulation of Treg with lower FOXP3 MFI and upregulation of Tbet. Overall, the authors found a reprogrammed Treg population that appears to be inflammatory rather than immunosuppressive associated with response to combination IO with anti‐PD1 + anti‐LAG3 in both mice and patients.

The findings from this study prompt several new sets of questions. First, it will be important to assess the degree to which human Treg in the tumor microenvironment mirror the phenotype of tumor infiltrating murine Treg. Second, a potential caveat was the use of subcutaneous tumor inoculation in the murine models, which is associated with differences in antitumor immunity versus orthotopic tumors [9, 10, 11]. Third, these findings build on previous studies in PBMC and suggest that this compartment plays an important role in dictating IO response in a subset of patients. As suggested by the authors, future prospective studies should elucidate whether there are PBMC biomarkers prior to IO that are predictive of initial resistance to anti‐PD1 monotherapy and suggestive of clinical benefit specifically to anti‐PD1 + anti‐LAG3 combination therapy (that is, the LAG3^hi^ immunotype). This prioritization would potentially save patients from being treated with a potentially inefficacious IO and would provide justification for combination therapy and its concomitant increased risk of immune related adverse events. Finally, it would be intriguing to determine whether this LAG3^hi^ immunotype is reflective of patients that will respond to anti‐PD1 + anti‐LAG3 in cancers beyond metastatic melanoma. Identification of a patient population earlier in the disease course that would benefit from combination IO would also be clinically impactful. Overall, Rolig, Peng and colleagues have demonstrated an important role for a population outside of CD8^+^ T cells in response to combination IO, which will help inform future combination IO strategies.

## Conflict of interest

TCB serves on the scientific advisory boards of Tabby Therapeutics, Tallac Therapeutics, Galvanize Therapeutics, Mestag Therapeutics, and Kalivir Therapeutics and is a consultant for Galvanize Therapeutics. ARC has no conflicts to declare.

## Author contributions

TCB and ARC jointly conceived and wrote this commentary.
